# Supplementary material for: Turnover of strain-level diversity modulates functional traits in the honeybee gut microbiome between nurses and foragers
Source: Genome Biol. 2023 Dec 8;24:283. doi: 10.1186/s13059-023-03131-4 (PMC10704631; doi:10.1186/s13059-023-03131-4)
Supplement: Supplementary file 1 — Additional file 1: Fig. S1. Per sample average gut mass, computed as each sample’s gut pool mass, divided by the number of guts in the pool. Fig. S2. (A) Stacked bar plot of the proportions of reads mapping to the bacterial genome database and to the A. mellifera genome, as well as non-mapped reads, for each of the samples. (B) Taxonomic profiling of the unmapped reads at the species-level with mOTU2. Colors correspond to genera. Percentages below each distribution show fraction of unmapped reads in each sample (also see panel A). Fig. S3. Stacked bar plots of the proportions of the different species and corresponding Shannon diversity index across nurse and forager samples for the four multi-species phylotypes (A) Bifidobacterium, (B) Lactobacillus, (C) Bombilactobacillus, and (D) Gilliamella. Fig S4. NMDS plot of the samples’ bacterial phylotype composition (A) and species composition (B). Samples are colored according to sampling location. PERMANOVA results: Phylotype-level, Location – R2=0.40279, p-value=0.001; Species-level, Location – R2=0. 40642, p-value=0.001.Fig. S5. Absolute abundance of the different species detected in the samples approximated by the normalized estimated number of 16S rRNA gene copies. Asterisks indicate q-values < 0.05. Fig. S6. Pearson correlation coefficient and non-corrected p-value for the correlation of the percentage of polymorphic sites per species with the coverage of the species normalized by the bacterial load for each sample. Fig. S7. Principal Coordinate Analyses of the Jaccard distance between all samples for the different species based on proportions of shared polymorphic sites. The color scheme shows information on the sampling sites of the different samples. The connecting lines indicates the two samples from the same colony. Fig. S8 Workflow used for the functional gene content analysis. Fig. S9. Genomic island of Gilli_2 encoding T6SS genes enriched in forager versus nurse samples. The genomic island is only present in [file 13059_2023_3131_MOESM1_ESM.pdf]

Supplementary Figures for

Turnover of strain-level diversity modulates functional traits in the honeybee gut microbiome between nurses and foragers

Gilles LC Baud, Aiswarya Prasad, Kirsten M Ellegaard, Philipp Engel

Department of Fundamental Microbiology, University of Lausanne, 1015 Lausanne, Switzerland

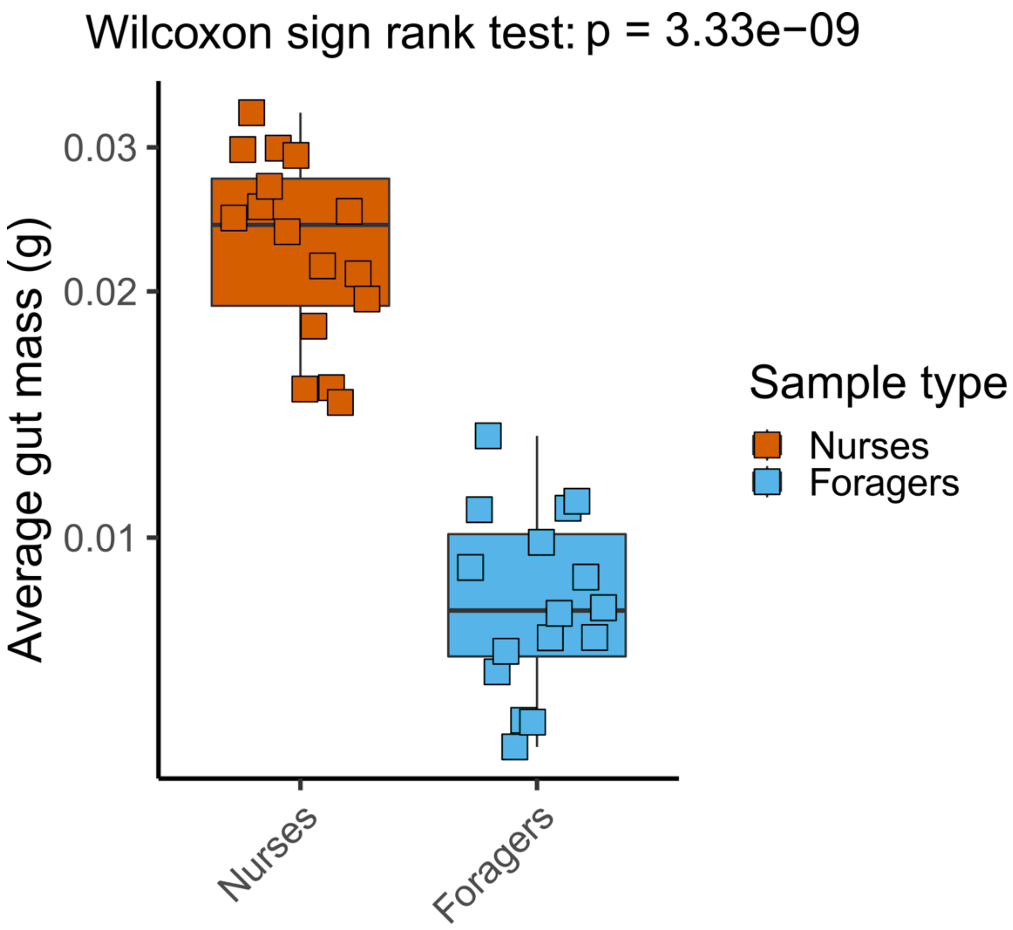

Fig. S1. Per sample average gut mass, computed as each sample's gut pool mass, divided by the number of guts in the pool.

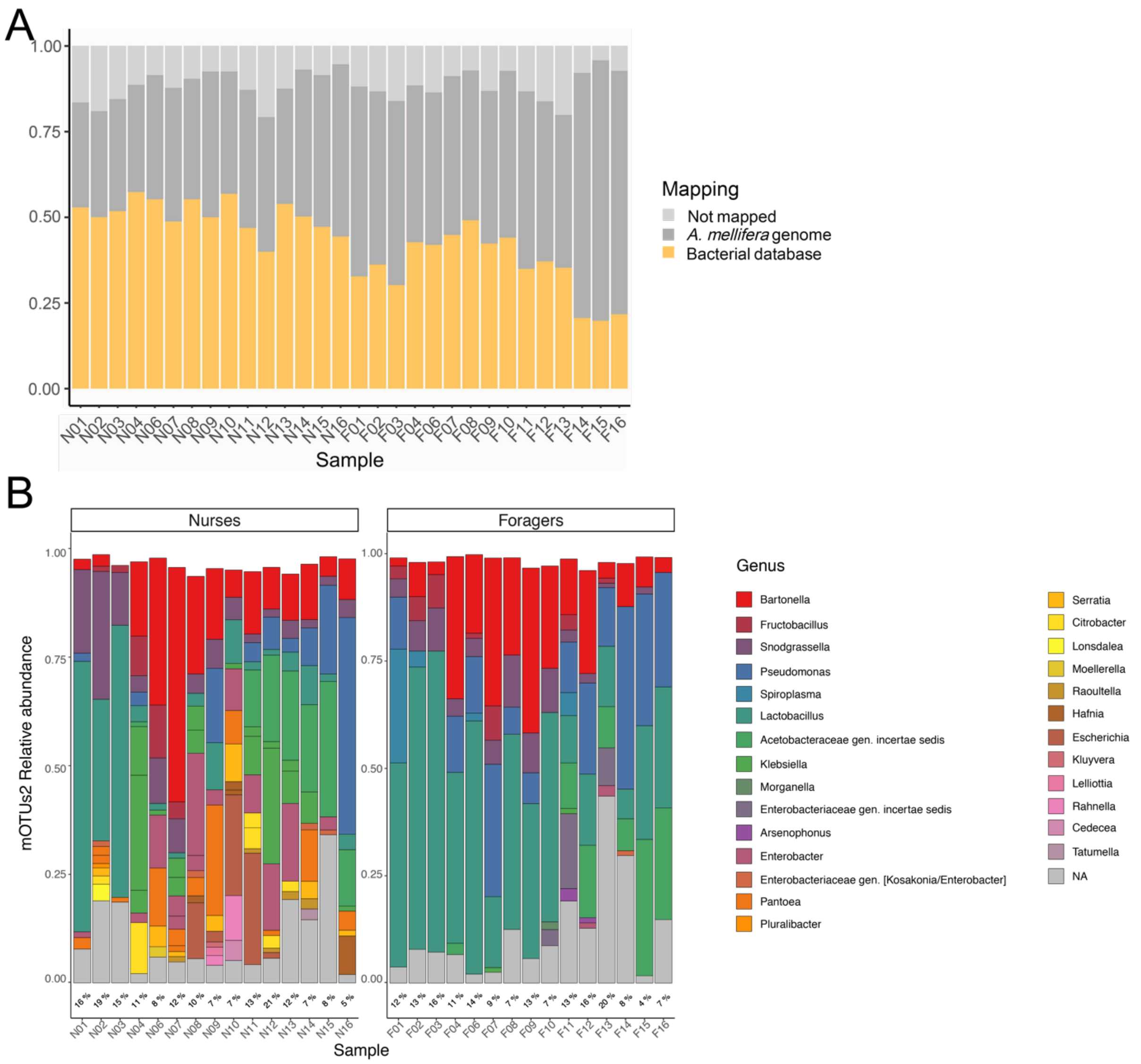

Fig. S2. (A) Stacked bar plot of the proportions of reads mapping to the bacterial genome database and to the *A. mellifera* genome, as well as non-mapped reads, for each of the samples. (B) Taxonomic profiling of the unmapped reads at the species-level with mOTU2. Colors correspond to genera. Percentages below each distribution show fraction of unmapped reads in each sample (also see panel A).

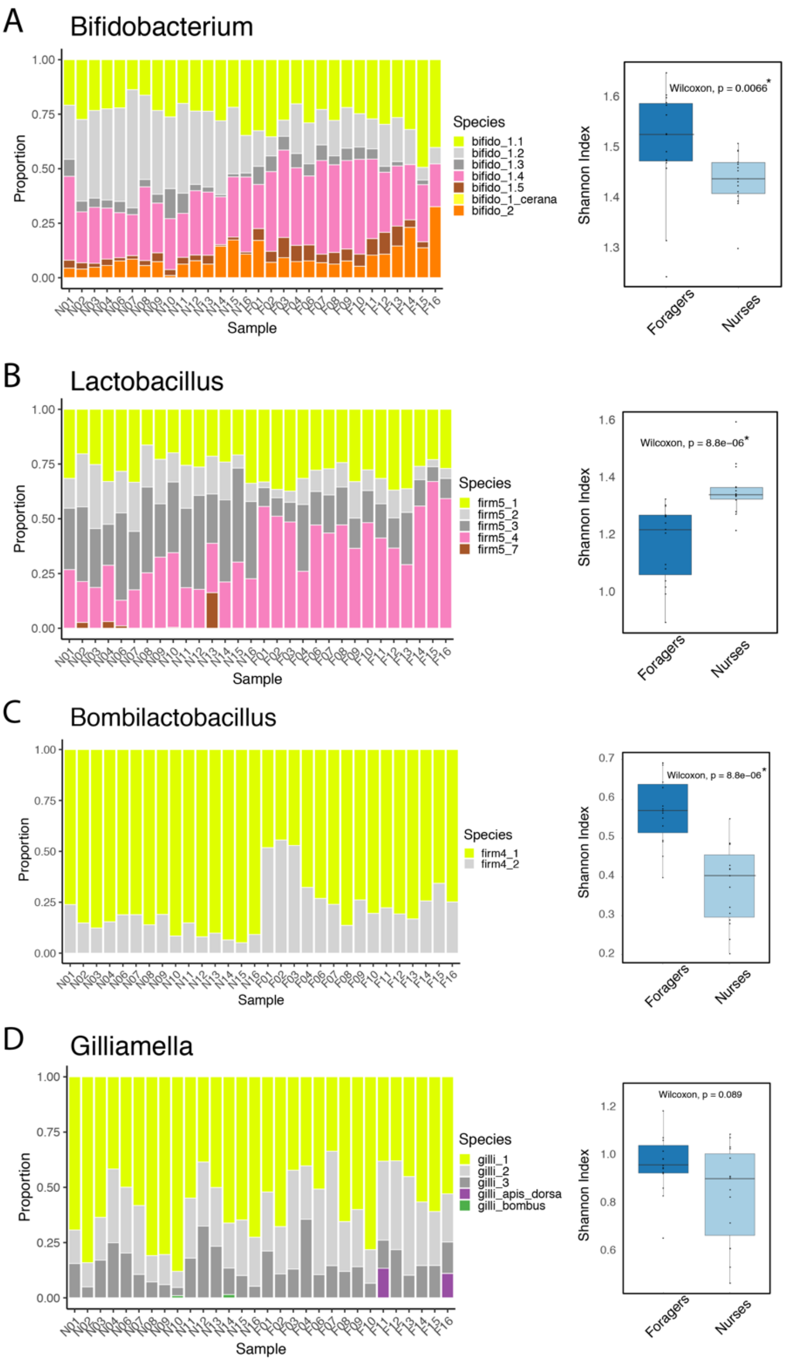

Fig. S3. Stacked bar plots of the proportions of the different species and corresponding Shannon diversity index across nurse and forager samples for the four multi-species phylotypes (A) Bifidobacterium, (B) Lactobacillus, (C) Bombilactobacillus, and (D) Gilliamella.

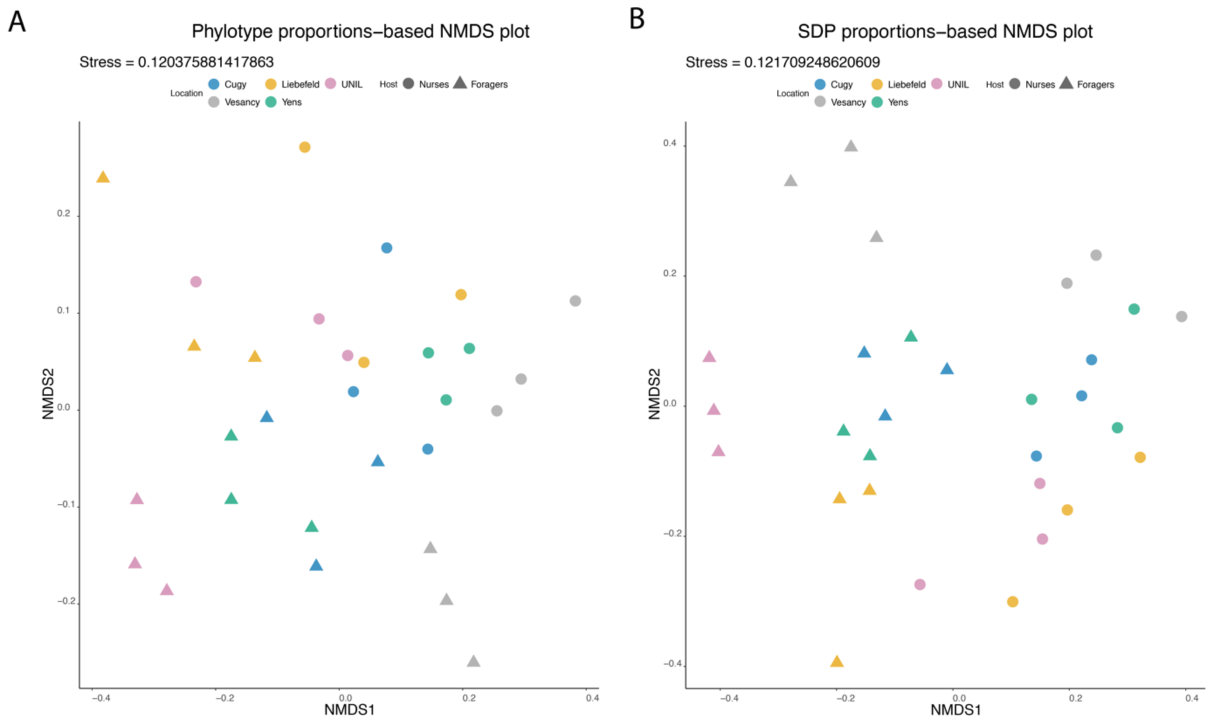

Fig. S4. NMDS plot of the samples' bacterial phylotype composition (A) and species composition (B). Samples are colored according to sampling location. PERMANOVA results: Phylotype-level, Location – R^2=0.40279, p-value=0.001; Species-level, Location – R^2=0. 40642, p-value=0.001.

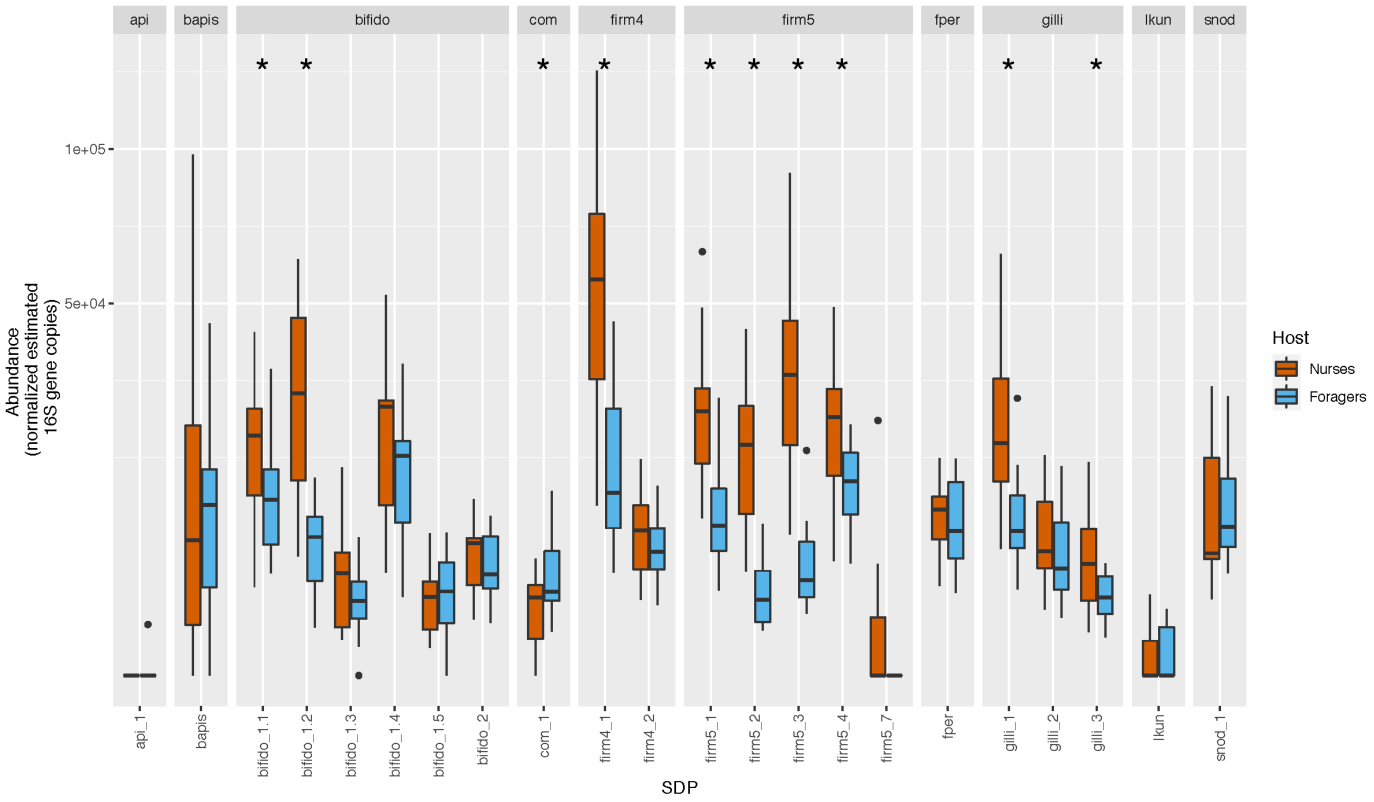

Fig. S5. Absolute abundance of the different species detected in the samples approximated by the normalized estimated number of 16S rRNA gene copies. Asterisks indicate q-values < 0.05.

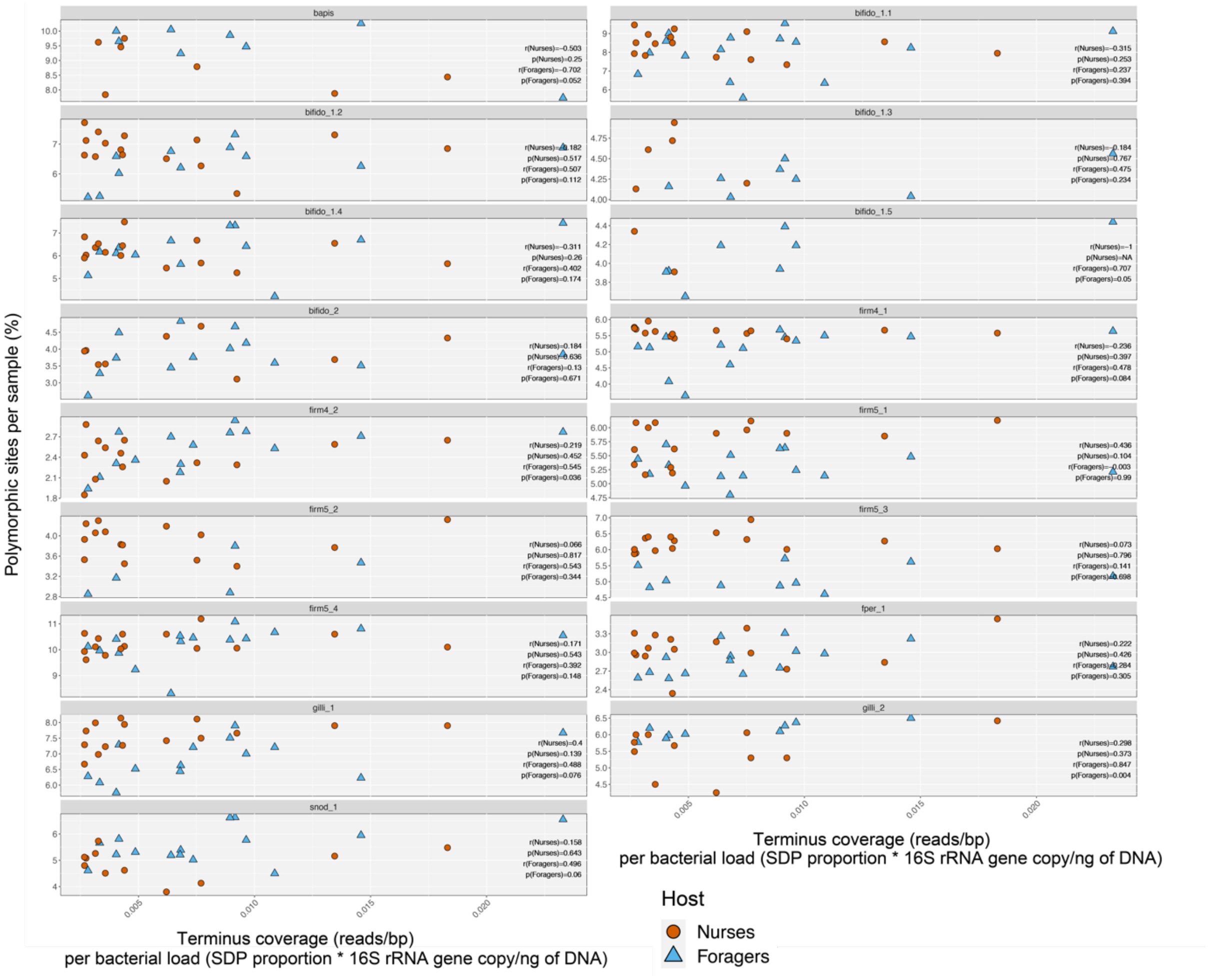

Fig. S6. Pearson correlation coefficient and non-corrected p-value for the correlation of the percentage of polymorphic sites per species with the coverage of the species normalized by the bacterial load for each sample.

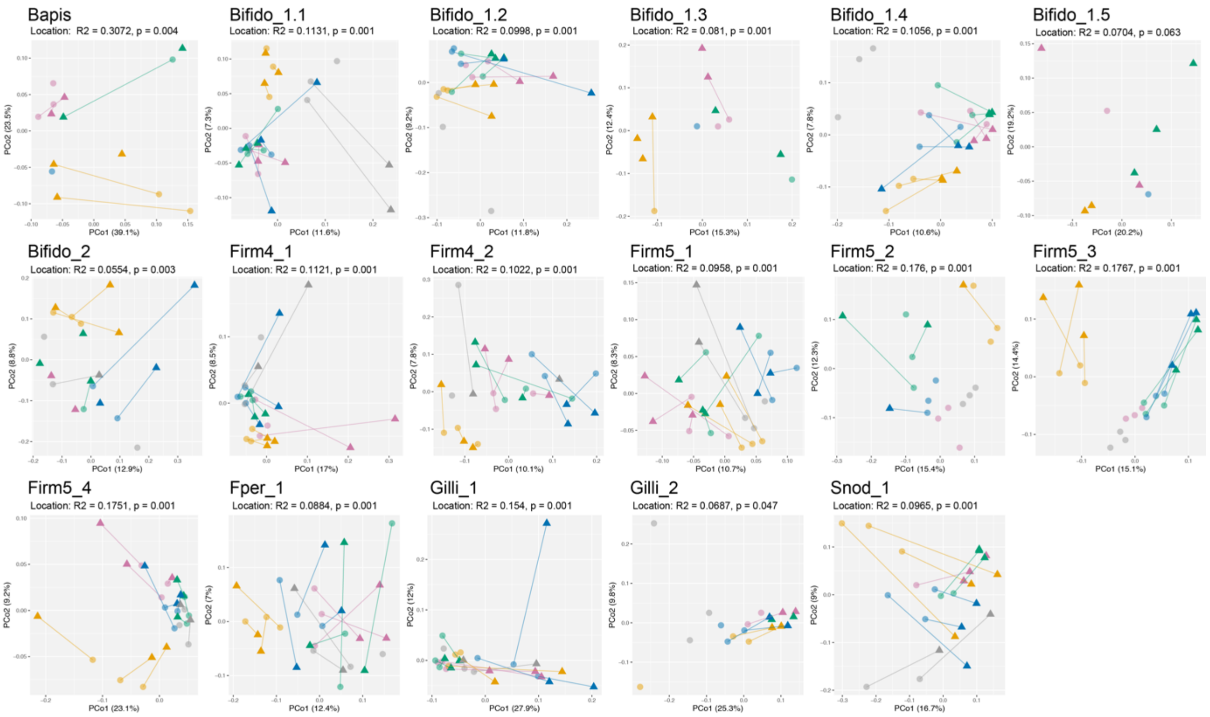

Fig. S7. Principal Coordinate Analyses of the Jaccard distance between all samples for the different species based on proportions of shared polymorphic sites. The color scheme shows information on the sampling sites of the different samples. The connecting lines indicates the two samples from the same colony.

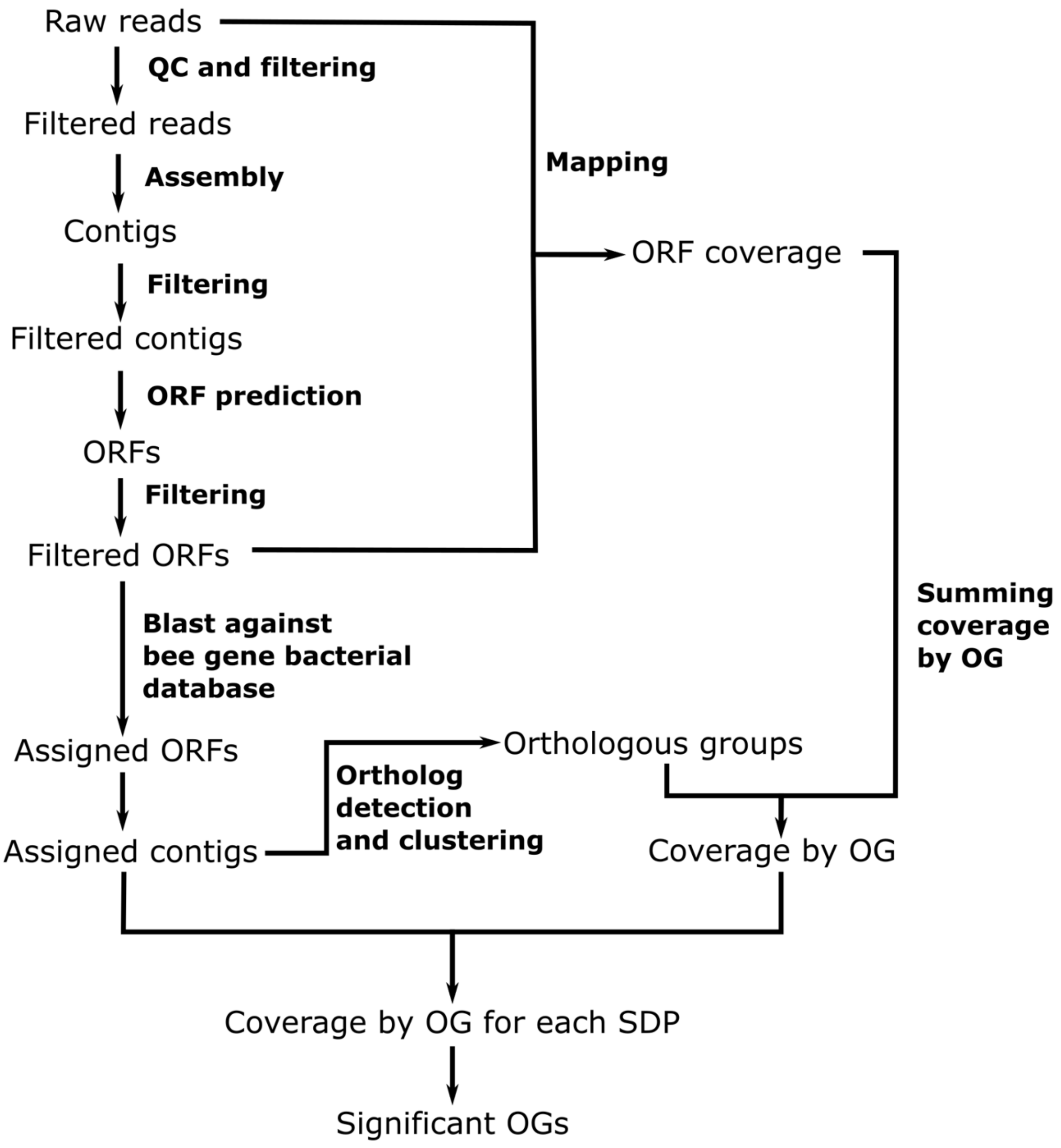

Fig. S8. Workflow used for the functional gene content analysis.

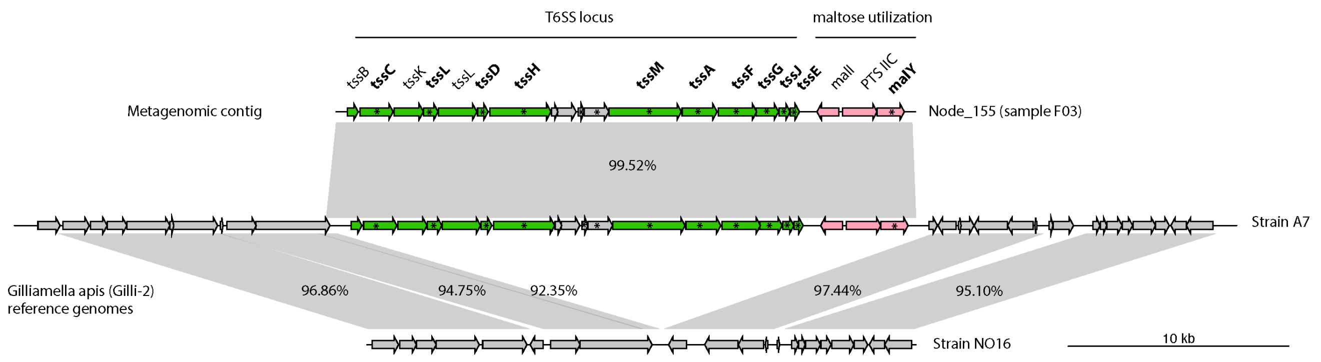

Fig. S9. Genomic island of Gilli_2 encoding T6SS genes enriched in forager versus nurse samples. The genomic island is only present in strain A7 of Gilli_2 in our genome database and is integrated in a conserved genomic region. Genes in green indicate T6SS genes, genes in pink depict strain-specific genes beloning to the same genomic island, and genes in grey are other other genes present in the flanking regions of genomes of Gilli_2. Genes enriched in forager samples are labeled in bold and flagged with an asterisk.

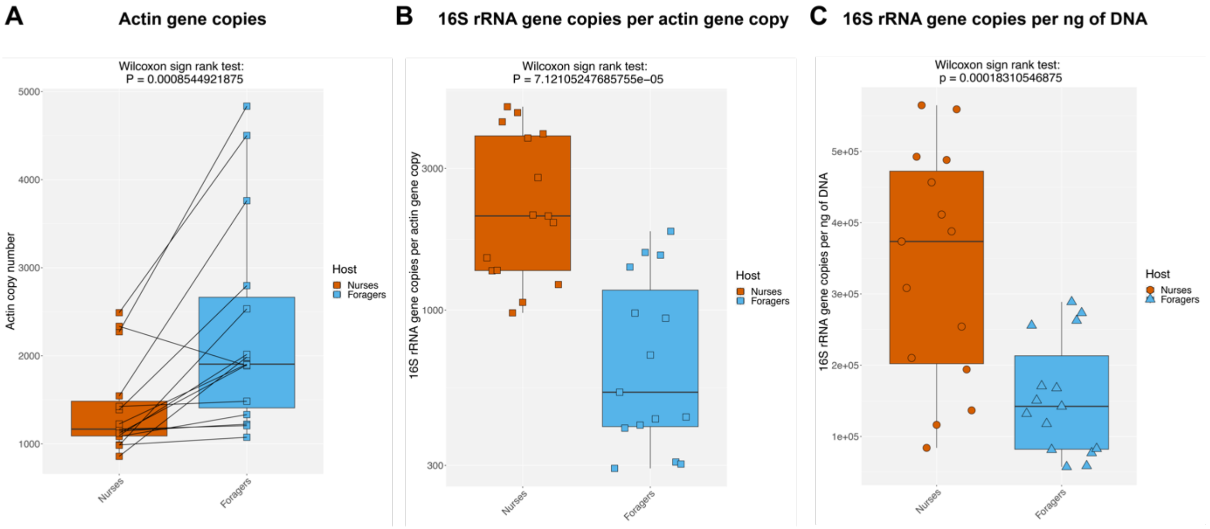

Fig. S10. (A) Actin gene copies in 10ng of each sample. (B) 16S rRNA gene copy number per actin gene copy per sample. (C) rRNA gene copy number per actin gene copy per ng of total DNA. The normalization with actin gene copies amplifies the differences between the two groups, as there are fewer copies in foragers than in nurses.

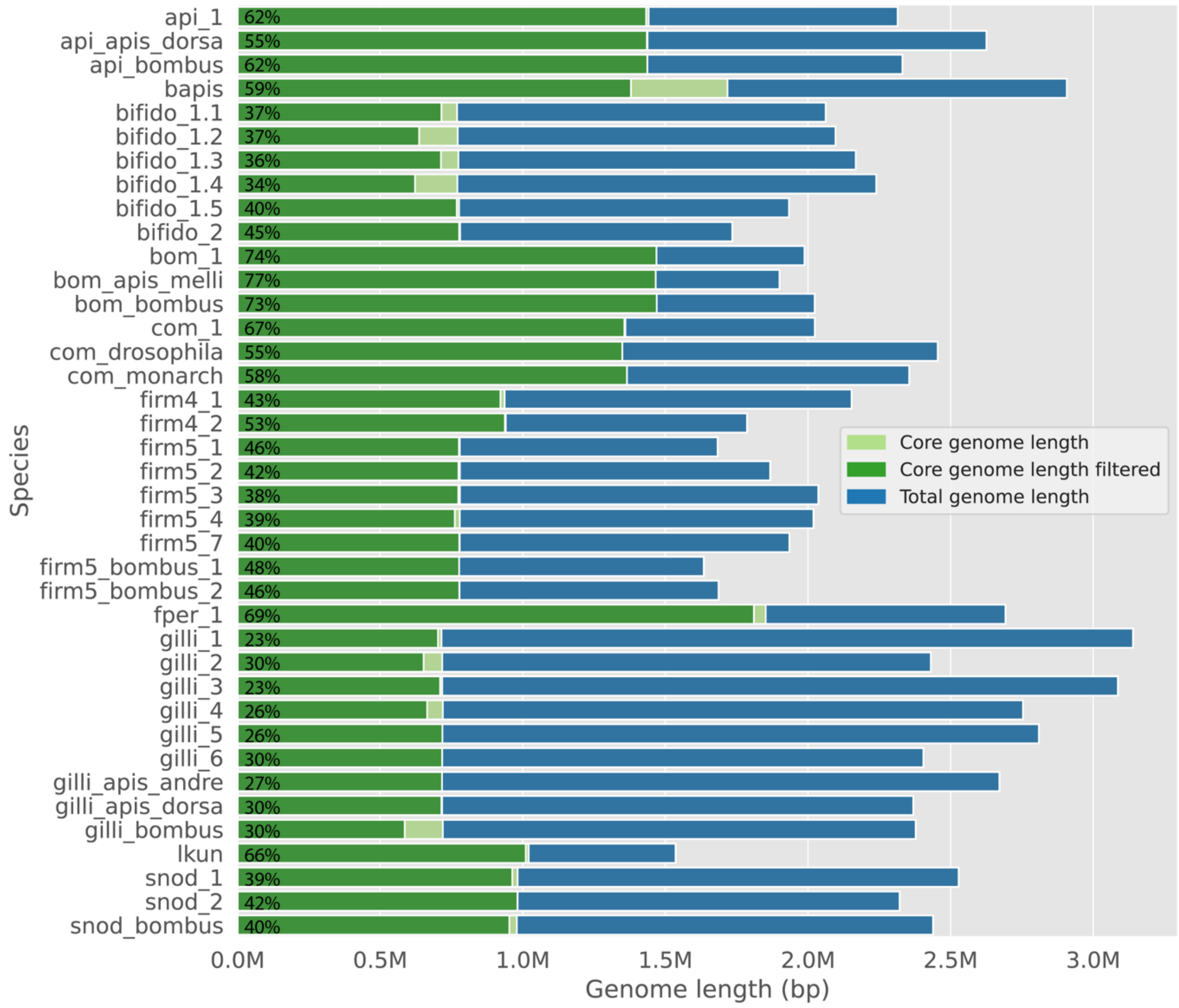

Fig. S11. Cumulative length of all single-copy core genes (Core genome length, in green) relative to the total genome length of the reference strain (Total genome length, in blue) of each species. Reference strain is the strain used in the reduced reference database. The filtered core genome length represents the portion of the core genes which were used for the SNV analysis as they had >10x read coverage across all samples.
